# Data on the TGFβ response of CD4^+^ T cells in the absence of *Eed*

**DOI:** 10.1016/j.dib.2018.02.045

**Published:** 2018-02-17

**Authors:** Taku Naito, Sawako Muroi, Ichiro Taniuchi, Motonari Kondo

**Affiliations:** a Department of Molecular Immunology, Toho University School of Medicine, Tokyo, Japan; b Laboratory for Gene Regulation, Institute for Medical Sciences, RIKEN, Yokohama, Japan

**Keywords:** TGFβ, PRC2, Eed, CD4^+^ CD8α^+^ T cells

## Abstract

The data presented here are related to the research article entitled “Loss of Eed leads to lineage instability and increased CD8 expression of mouse CD4^+^ T cells upon TGFβ signaling” [1]. The cited research article investigates the molecular mechanism of CD8α upregulation observed in *Eed*-deficient (*∆Eed*) CD4^+^ T cells upon activation in the presence of TGFβ. This data report describes the effect of retinoic acid (RA) and/or anti-interferon-gamma (IFNγ) antibody supplementation on up-regulation of CD8α and Foxp3 in *∆Eed* CD4^+^ T cells, the effect of dose or timing of TGFβ treatment on CD4^+^ T cell identity of *∆Eed*, adding further information regarding the conditions that induces CD8α, and mRNA expression changes of genes encoding polycomb repressive complex 2 (PRC2) subunits by TGFβ treatment.

**Specifications Table**

**Table:** 

Subject area	*Immunology and Molecular Biology*
More specific subject area	*Differentiation of T-helper subsets*
Type of data	*Graphs and flow cytometry plots.*
How data were acquired	– *Flow cytometry (FACSCanto II and FACSAria III, BD Biosciences)* – *Quantitative PCR (qPCR) (ABI 7500 Fast, ABI and QuantStudio3, Thermo Fisher)*
Data format	*Analyzed*
Experimental factors	– *The Eed gene was specifically deleted in T cells by crossing mice carrying the floxed Eed alleles* [1] *with the CD4-Cre transgenic mouse* [2]. – *Naïve (CD25^-^ CD62L^high^ CD44^low^) CD4*^*+*^ *T cells of wild type or ∆Eed mouse were cultured in the presence of anti-CD3/anti-CD28 supplemented with cytokines, neutralizing antibodies, and/or retinoic acid.* – *RNA samples were recovered using TriZOL (Life Technologies), reverse transcribed using Super Scirpt III (TaKaRa) with oligo-dT primers, and then subjected to qPCR analysis using ExTaq II SYBR Green Reagent (TaKaRa).* – *Expression of proteins and cell surface markers was assessed by flow cytometry.* – *Various concentrations or timings of TGFβ treatment were tested.*
Experimental features	– *Expression of Foxp3 in activated wild type or ∆Eed T cells treated with TGFβ, RA and anti-IFNγ antibody.* – *Expression of CD4 and CD8α on ∆Eed T cells treated with varying doses, or differing time windows, of TGFβ.* – *Expression of genes encoding PRC2 subunits after TGFβ treatment.*
Data source location	*Toho University School of Medicine, Tokyo, Japan*
Data accessibility	*Data are within this article.*

**Value of the data**

• The data show the limited capability of *∆Eed* CD4^+^ T cells to differentiate toward the Foxp3^+^ Treg lineage by TGFβ treatment, regardless of the presence of RA or the inhibition of IFNγ.
• The data reveal how *∆Eed* CD4^+^ T cells upregulate CD8α in response to different dose of TGFβ, or how the addition or withdrawal of TGFβ at different time points affects CD8α induction of *∆Eed* CD4^+^ T cells.
• The data show that the mRNA levels of *Eed* and *Ezh2* changes in the presence of TGFβ upon activation.

## 1 Data

**Fig. 1:**
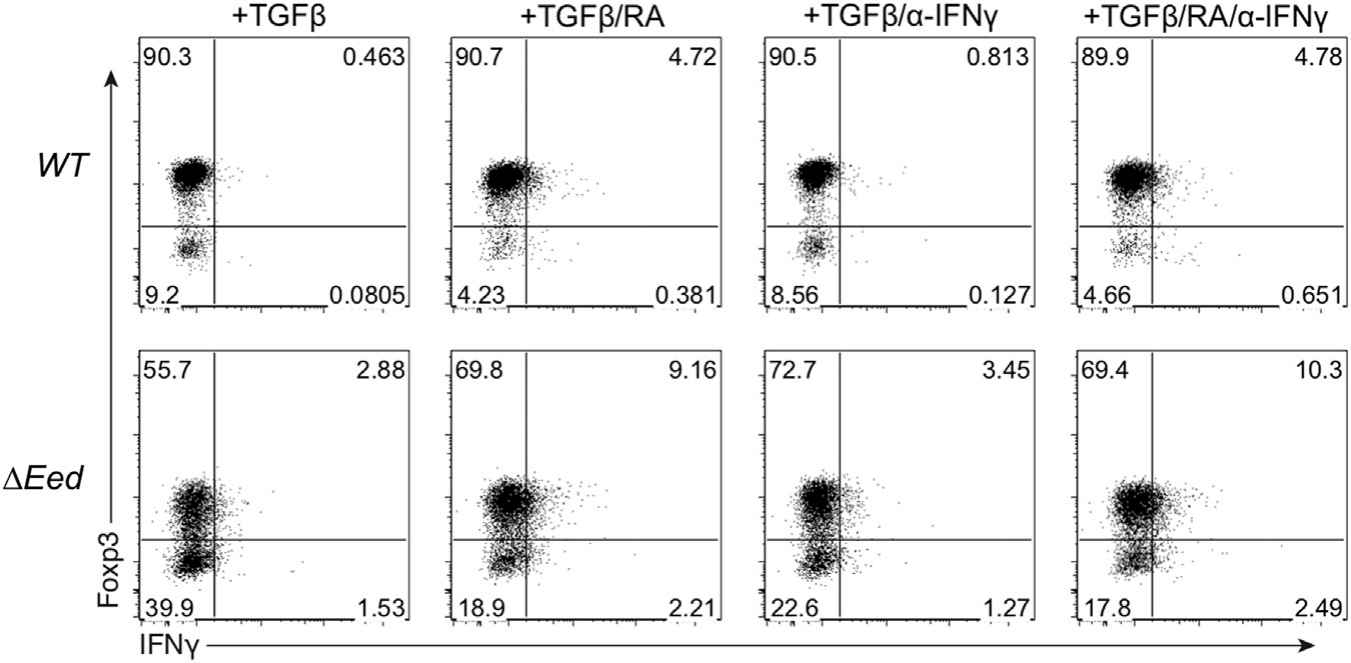
Effects of RA and anti-IFNγ antibody on Treg differentiation in *∆Eed* T cells. T cells were activated with anti-CD3/anti-CD28 antibody in the presence of TGFβ, supplemented with RA, anti-IFNγ antibody, or both. Profiles of Foxp3 and IFNγ expression under each condition are shown.

**Fig. 2:**
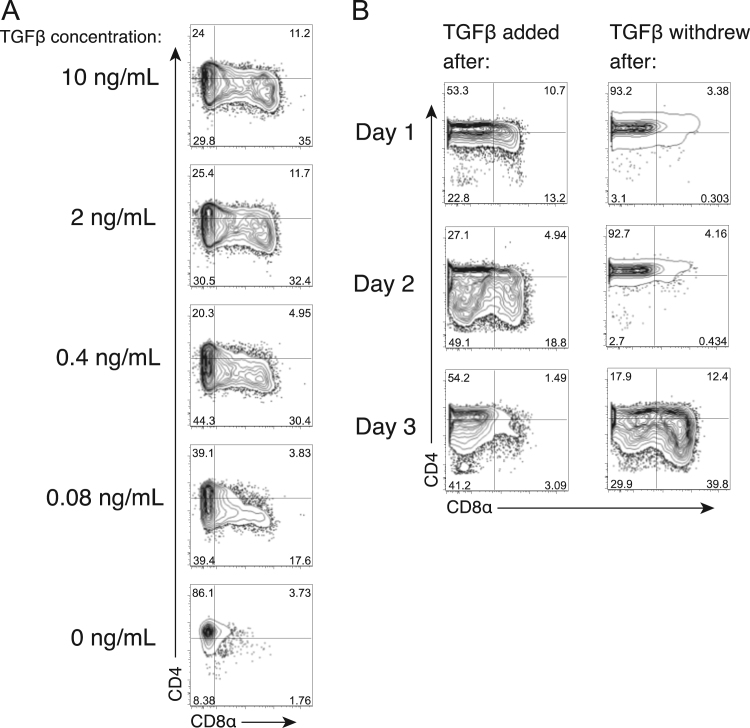
Effect of TGFβ concentration and timing on CD8α induction. (A) *∆Eed* T cells were activated with anti-CD3/anti-CD28 antibodies in the presence of the indicated amount of TGFβ. The CD4/CD8α profiles of 6 days post-activation are shown. (B) *∆Eed* T cells were activated with anti-CD3/anti-CD28. Ten ng/mL of TGFβ was added to the culture one, two or three days after activation (left column). Alternatively, TGFβ was included in the culture at the time of activation and then withdrew one, two or three days after activation (right column). The CD4/CD8α expression profiles of 6 days post-activation are shown.

**Fig. 3:**
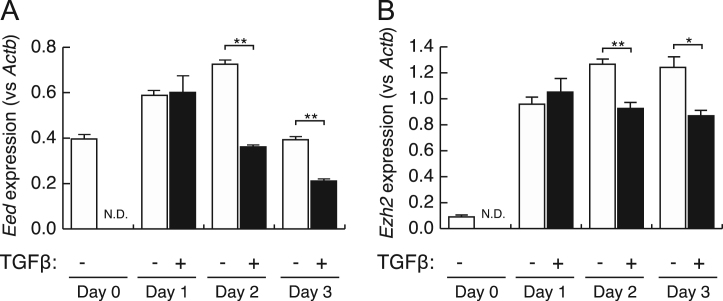
Expression changes of PRC2 component by TGFβ treatment. Naïve *WT* CD4^+^ cells were activated with anti-CD3/anti-CD28 in the presence or absence of TGFβ, and expression of *Eed* or *Ezh2* was examined by qPCR, normalized by *Actb* expression. Mean ± S.E.M. are shown. *P < 0.05 and **P < 0.01 by Student *t*-test. N.D.: no data.

The expression of Foxp3 in TGFβ-stimulated wild type and *∆Eed* CD4^+^ cells in the presence of RA and/or anti-IFNγ are shown in Fig. 1. The response of *∆Eed* CD4^+^ T cells to different concentrations of TGFβ, or to TGFβ added in different time windows after anti-CD3/anti-CD28 activation, are shown in Fig. 2. The changes in the expression level of *Eed* and *Ezh2* after anti-CD3/anti-CD28 activation in the presence or absence of TGFβ, measured by qPCR, are presented in Fig. 3.

## 2 Experimental design, materials and methods

### 2.1 Cell isolation, cell culture, and flow cytometry

Detailed procedures and used reagents are as previously described [1].

### 2.2 Quantitative PCR

Detailed procedures of RNA isolation, cDNA synthesis and qPCR are as previously described [1]. The sequences of primers used in this study are: Eed forward; gttgagcagcgacgagaacag, Eed reverse; gtgccactctcaatactgacag, Ezh2 forward; actgctggcaccgtctgatg, Ezh2 reverse; tcctgagaaataatctccccacag.

### 2.3 Statistical analysis

Data were analyzed using a two-tailed, paired Student *t*-test where appropriate.
