# Supplementary material for: Data on the TGFβ response of CD4+ T cells in the absence of Eed
Source: Data Brief. 2018 Feb 17;17:1180–3. doi: 10.1016/j.dib.2018.02.045 (PMC5988442; doi:10.1016/j.dib.2018.02.045)
Supplement: Supplementary file 1 — Supplementary material [file mmc1.docx]

Conflict of Interest Statement

The authors have no conflict of interest to disclose.
